# UV Laser Micromachining of FR-4-Based Rigid–Flex PCBs: Predictive Modeling of Penetration Depth Through Design of Experiments

**DOI:** 10.3390/mi17030351

**Published:** 2026-03-13

**Authors:** Giorgio Pellei, Paolo Di Stefano, Luca Mascalchi, Renzo Centi

**Affiliations:** 1 Department of Industrial and Computer Engineering and Economics, University of L’Aquila, 67100 L’Aquila, Italy; paolo.distefano@univaq.it; 2 Elco S.p.A., 67061 Carsoli, Italy; luca.mascalchi@elcopcb.com (L.M.); renzo.centi@elcopcb.com (R.C.)

**Keywords:** UV laser micromachining, rigid–flex PCBs, design of experiments, predictive modeling, penetration depth, FR-4, material removal, process optimization

## Abstract

This study developed predictive mathematical models for UV laser penetration depth in FR-4-based rigid–flex printed circuit boards, addressing the critical need for precise material removal in applications like protective plug removal. Utilizing a comprehensive Design of Experiments framework, specifically two-level full factorial designs, the influence of key operational parameters—number of loops, scanning speed, and focal position offset—on material removal was systematically investigated in both laminate and multilayer substrates. Empirical models were established for both substrate types, identifying significant factors and interactions that govern penetration depth with physical justification. Comparative analysis revealed that the multilayer model consistently predicted deeper penetration (6–17 µm) than the laminate model under identical conditions, primarily due to reduced heat-associated phenomena with prepreg, yet the laminate model offered a reasonable approximation for complex stack-ups. Rigorous validation through confirmation experiments, achieving 100% success in electrical integrity tests with compliant plug removal, unequivocally demonstrated the models’ robustness and reliability. This research provided a crucial tool for optimizing UV laser micromachining processes, significantly reducing parameter identification times and minimizing scrap generation, thereby enhancing the efficiency and reliability of advanced rigid–flex PCB manufacturing.

## 1. Introduction

Laser micromachining processes have garnered substantial attention across the electronics industry, particularly for rigid–flex printed circuit boards, owing to their capacity for precise material removal with minimal thermal damage [1]. This precision is critical in applications demanding high dimensional tolerances, such as decapping [2], micro-channel fabrication, and micro-via drilling [3]. The intricate design of rigid–flex PCBs, which integrate both flexible and rigid sections, necessitates exceptionally accurate processing to preserve the integrity of their delicate interconnections [1].

Ultraviolet laser micromachining has emerged as a highly viable solution for material removal in sensitive electronic components. Its advantages stem from a minimal thermal impact due to the short pulse durations and specific wavelengths, which limit thermal diffusion and the associated spread of the heat-affected zone, and its high precision [1,4]. This enables cleaner cuts and superior control over ablation depth in various materials, including FR-4 and copper [5,6]. Furthermore, UV lasers are effective in ablating both the matrix and fibrous components of composite materials like FR-4 [7], offering versatility unmatched by other laser types. The process can be effectively optimized through Design of Experiments methodologies, which facilitate the identification of optimal processing windows [8,9]. Predictive modeling utilizing DoE directly addresses the need for efficient parameter optimization in complex laser micromachining applications involving multiple interacting variables by minimizing the number of required experimental trials [10,11].

The systematic analysis of critical process parameters—such as the number of loops, scanning speed, and focal position offset—through response surface methodology, a key component of DoE, enables the establishment of robust predictive correlations for penetration depth in multilayer substrates [12,13]. Optimizing the interaction between the UV laser source and the heterogeneous layers of rigid–flex PCBs, which may include FR-4 laminate, copper cladding, and prepreg layers, requires a rigorous experimental approach to establish reliable regression models for depth predictions [14]. This is particularly challenging given the distinct ablation mechanisms that can occur, ranging from photochemical to photothermal depending on the laser energy density and material properties [15]. Consequently, this study aims to develop empirical models correlating these parameters with material removal depth in multilayer composite substrates, specifically to enhance process control, focusing on the removal of protective plugs from the flex sections of PCBs. The paper details the application of ultraviolet laser systems and Design of Experiments methodologies to optimize material removal processes for multilayered rigid–flex PCB substrates.

This experimental activity was carried out in collaboration with Elco S.p.A., a PCB production factory based in Italy, and, due to industrial secrecy, the exact equations of the models cannot be disclosed, but their generalized form—which incorporates linear and interaction terms for the input parameters—can be presented. However, the removal of protective plugs in rigid–flex PCBs represents a critical micromachining step, where insufficient selectivity may result in incomplete removal, while excessive energy leads to damage to the flexible layers or thermal degradation. Despite its industrial relevance, quantitative process models for UV laser-based plug removal are still limited. For this reason, it was necessary to propose a DoE-based predictive framework to define a robust and selective process window for UV laser micromachining of rigid–flex PCBs, enabling controlled material removal. The proposed predictive models are intended for process optimization and as a tool to identify a stable and selective micromachining window rather than as a transferable numerical recipe. Therefore, the discussion focuses on process trends, parameter interactions, and robustness. Thanks to this experimental activity, it was possible to substantially reduced the time required to identify optimal parameters for rigid–flex PCB micromachining, while minimizing the risk of scrap generation due to poor parameter choices.

## 2. Materials and Methods

The experimental approach employed a Design of Experiments framework—specifically employing full factorial designs—to systematically investigate the influence of key laser processing parameters on penetration depth in both laminate and multilayer rigid–flex substrates [16,17]. These parameters—including the number of passes, scanning speed, and focal position—were systematically varied to establish their individual and interactive effects on ablation depth [12]. Statistical analysis used regression equations to quantify the relationships between these independent variables and penetration depth, drawing on established methods for non-linear statistical models in laser ablation processes. Empirical data for the experimental validation of these predictive correlations were generated on FR-4-based laminate and prepreg composite structures [17].

Parameter ranges were determined from preliminary trials to ensure effective material ablation without substrate damage from excessive energy deposition. Notably, exceeding 15 consecutive passes increased heat damage and charring, degrading ablated feature quality and raising the risk of delamination and open or short circuits in the PCB’s internal circuitry.

A fixed power of 5.2 W was also used, as it represents the optimal working point for the UV laser source, ensuring beam stability and quality. Scanning speed was determined within the range of 350–450 mm/s; lower speeds lead to excessive carbonization, while higher speeds result in excessively long processing times. The focal position offset was not set below ∆z= −0.4 mm to avoid an excessively out-of-focus UV laser beam. The specific materials and methodology used in these experiments are detailed in the subsequent sections.

While this study focused on the operational parameters of loops, speed, and focal offset, it is critical to acknowledge the fundamental roles of pulse energy and fluence in the ablation process. In this experiment, the average power (*P*) was maintained constant, as was the pulse repetition frequency (f), effectively fixing the pulse energy according to the relation $E_{p}=P/f$ [18].

From a physical perspective, material removal is only initiated when the laser fluence—the energy delivered per unit area ($J/{cm}^{2}$)—exceeds the specific ablation threshold of the FR-4 composite [19,20]. The decision to fix $E_{p}$ establishes a stable energy regime; however, the effective fluence on the material surface is dynamically modified by the focal position offset (Δz).

Regarding the generalizability of the developed models, these equations are specifically calibrated for the fixed pulse energy and frequency used in this study, which are representative of the majority of operating laser systems in the PCB industry. Should the average power or repetition rate be altered, the resulting change in pulse energy would shift the ablation efficiency and potentially the dominant mechanism (e.g., from photochemical to photothermal) [21]. Consequently, while the identified interactions between loops, speed, and focus remain qualitatively relevant, the quantitative depth predictions would require recalibration if the laser’s fundamental energy parameters were significantly adjusted.

- Laser cutting machine:

A Laser Combi Drill 350 by Schmoll Maschinen (Rödermark, Germany) was utilized, equipped with dual laser sources: a UV laser for copper removal and hole cleaning and a CO_2_ laser for dielectric removal.

For the UV laser, a Coherent AVIA LX Q-switched diode-pumped nanosecond laser was used. The laser wavelength was 355 nm. The laser frequency was set at 100 kHz. The pulse duration of the laser beam was set at 30 ns. The output beam profile was Gaussian-shaped. The beam divergence was less than 0.2 mrad. The spatial mode was TEM_00_ (M^2^ = 1.2). A laser beam with a diameter of 3.0 mm (at 1/e^2^) was introduced to the PCB substrate using a galvanometric scanner with an F-Theta lens achieving a spot size of 18.08 μm (1/e^2^). The focal length was 100 mm.

- Laminate:

A double-sided laminate with a 1.6 mm dielectric thickness, where both top and bottom copper clads had been removed by etching, was selected to evaluate the interaction between the UV laser and FR-4 materials and then transpose these findings to a production plug removal operation through UV laser ablation. For this purpose, the resulting model was then compared with one derived from multilayer substrates consisting of prepreg and laminate stacks to assess the impact of increased structural complexity on ablation characteristics.

**Figure 1:**
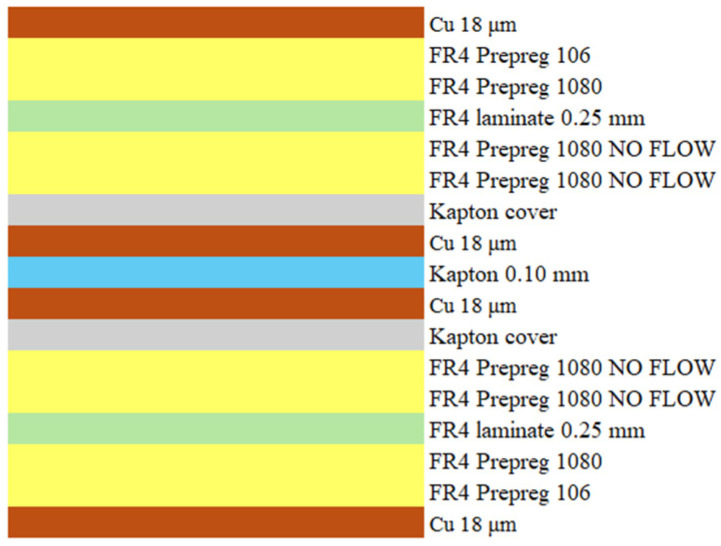
Multilayer substrate used for the experimentation.

- Multilayer substrate:

This consisted of alternating layers of 1080 and 106 prepreg interleaved with FR-4 laminates, as shown in Figure 1.

The protective cap has a total thickness of 490 µm and consists entirely of dielectric material. This setup simulated a real production rigid–flex protective plug, ensuring that the experimental conditions accurately replicate the thermal and mechanical phenomena encountered during actual plug removal operations on rigid–flex PCBs.

- Test coupon:

The specimen extracted from the panel was a 20 mm × 20 mm grid containing 10 cuts, each representing a specific DoE condition. To account for inherent material and process variability—such as inconsistencies in laminate/prepreg composition and laser beam fluctuations—thereby enhancing the predictive model’s robustness and generalizability to industrial production, each test coupon grid was fabricated across the panel and extracted from a random position.

- Grinding machines and resins:

For metallographic sectioning, the specimen underwent embedding and lapping operations. Embedding was performed using cold-curing methyl methacrylate resin 609 produced by LAM PLAN (Gaillard, France). For the subsequent grinding and polishing steps, PRESI Mecapol P255U2 and PRESI Mecatch 250 (Eybens, France) equipment, along with various abrasive films and suspensions, was employed to achieve the requisite surface finish for microscopic analysis.

- Optical microscope:

Sample analysis was conducted using the Leica DM 2500M (Wetzlar, Germany) optical microscope. This instrument, suitable for material analysis and equipped with dedicated Leica software (Wetzlar, Germany), enabled the examination of metallographic sections and acquisition of the desired measurements.

- Software:

Design-Expert^®^ software (version 13, Stat-Ease Inc., Minneapolis, MN, USA) was utilized for the statistical Design of Experiments, data analysis, and the development of the predictive mathematical models. This software facilitated the implementation of the two-level full factorial designs, enabling the systematic investigation of parameter effects, ANOVA analysis to determine statistical significance, and the generation of regression equations for penetration depth.

- Design of Experiment (DoE):

To systematically assess the effects of key UV laser parameters on penetration depth in both laminate and multilayer structures, a Design of Experiments using two-level full factorial designs was implemented. This approach enabled comprehensive analysis of the individual and interactive effects of these parameters—specifically, the number of loops, scanning speed, and focal position—on cut penetration depth in the substrates. Only parameters with p<0.05 were included in the final model.

**Figure 2:**
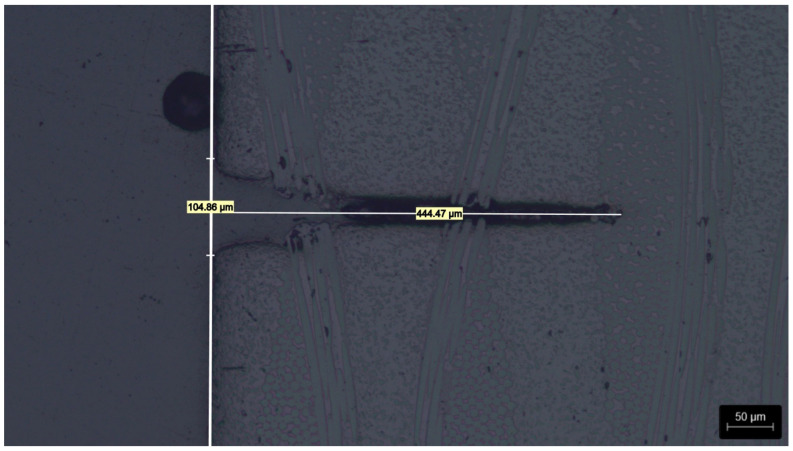
Example of UV laser penetration depth measurement.

- Measuring approach of the cross-sections:

For each DoE condition, five cuts were analyzed to ensure statistical significance, with their average depths used as the response outputs (Figure 2). Predictive models for UV laser penetration depth ($h_{p}$) were then developed for both the laminate and multilayer substrates.

## 3. Results and Discussion

In this section, we present the results derived from the DoE, focusing on the statistical significance of the investigated parameters and their implications for predicting UV laser penetration depth across FR-4 laminate and multilayer assemblies. Specifically, this analysis contributes to the formulation of predictive mathematical functions that correlate operational parameters—such as the number of loops, scanning speed, and focal position—with the resulting ablation depth in the studied substrates.

### 3.1. Laminate Substrate

The experimental conditions of the 2^3^ full factorial DoE are presented in Table 1.

To clarify the definition of focal position offset, it is quantified as the offset ∆z between the nominal focal position (z = 0) and the actual set position. A negative ∆z positions the UV laser head below the nominal focus (closer to the workpiece substrate), producing a divergent beam with a larger spot size. A value of ∆z=0 indicates operation at the precise focal position.

Then, as described previously, for each DoE condition, five cuts were measured to ensure statistically significant results, and their means were used as the DoE response outputs, as shown in the following Table 2.

**Figure 3:**
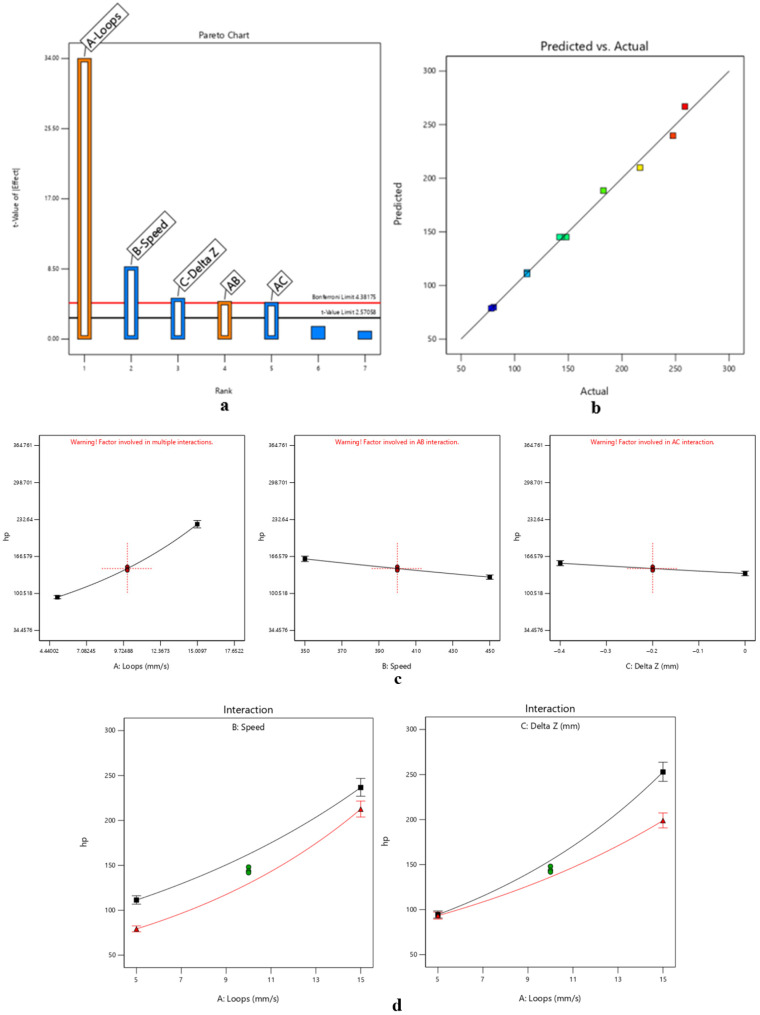
DoE-based analysis of UV laser plug removal in rigid–flex PCBs simulated with FR-4 laminate, highlighting (**a**) dominant parameters affecting removal selectivity, (**b**–**d**) process trends defining the stable micromachining window.

ANOVA was performed to assess the significance of the model terms, yielding the following results detailed in Table 3 and Figure 3.

The obtained model for hp can be expressed as the base-10 logarithmic law of the form ${log}_{10}(h_{p})$, where the statistically significant factors are:

- Loops, with a positive effect on the response.
- Speed, with a negative effect on the response.
- Focal position offset, with a negative effect on the response.
- Loops–speed interaction, with a positive effect on the response.
- Loops–focal position interaction, with a negative effect on the response.

Based on the ANOVA results, speed–focal position interactions and third-order interactions were found to be statistically insignificant for predicting penetration depth (*p* > 0.05) and thus were excluded from the final predictive model. It is interesting to observe that the most influent factor is the number of loops, which contributes 88.81% to the total variance in penetration depth; the other parameters and interactions contribute to a lesser extent (<6%) but still significantly influence the ablation process.

This reduced model, which focuses only on the significant factors, provides a robust mathematical equation for predicting penetration depth. Its validity is confirmed by the model’s very low *p*-value, the absence of a lack of fit and the high value of $R_{pred}^{2}$.

Additionally, the adoption of a base-10 logarithmic transformation for the penetration depth is not only statistically advantageous for stabilizing variance but is also deeply rooted in the physics of laser–matter interaction. Fundamentally, the absorption of laser energy within a medium follows the Beer–Lambert Law, which dictates that light intensity diminishes exponentially with depth; consequently, the ablation depth typically scales logarithmically with the applied energy density or fluence [15,20].

Mechanistically, as the number of loops increases and the cut deepens, the process enters a regime of depth saturation. This is caused by several non-linear factors: the increasing aspect ratio leads to significant beam divergence at the ablation front, and the accumulation of an ablation plume (debris and plasma) partially shields the target from subsequent pulses [22,23]. Furthermore, in composite materials like FR-4, the incubation effect—where previous pulses lower the ablation threshold of the resin—follows a non-linear trend that is effectively linearized by a logarithmic scale [21,24]. Therefore, the logarithmic model accurately captures the ‘diminishing returns’ in penetration depth observed as cumulative energy deposition increases over multiple passes [20,25].

The generalized form of the model equation is:

(1) $${log}_{10}\left(h_{p}\right)=\alpha_{0}+\alpha_{1}*A-\alpha_{2}*B-\alpha_{3}*C+\alpha_{3}*AB-\alpha_{4}*AC$$

These parameters play a key role in defining the selective removal of the protective plug: an insufficient number of loops leads to an incomplete plug removal, while an excessive number of loops leads to penetration into the flexible layer, resulting in its irreversible damage. Analogously, an excessively high scanning speed or an improper focal position offset results in insufficient energy density for complete ablation, leaving residual material that compromises the plug removal process, or, conversely, in a potentially catastrophic over-penetration that damages the underlying flexible circuitry.

The loops–speed interaction suggests that the penetration depth gain from increasing the number of loops is attenuated at higher scanning speeds due to the reduced energy dwell time per unit area, thereby mitigating excessive depth increases from additional passes.

Similarly, the loops–focal position interaction demonstrates the adverse impact of focal offset becoming more pronounced at higher loop counts. This necessitates stringent focal control to avert over-penetration into the flexible layer or incomplete ablation of the protective plug, aligning with the model’s emphasis on loops as the dominant factor.

Consequently, the developed predictive model enables precise control over the ablation depth, allowing operators to optimize the process parameters to achieve complete plug removal while preserving the integrity of the flexible circuit.

These considerations also confirm the physical justification of the laminate model: increasing the number of loops leads to deeper penetration, while higher scanning speeds and focal position offsets yield shallower penetration.

To ensure the adequacy of the developed predictive models and the validity of the ANOVA results, the underlying statistical assumptions were verified through residual analysis, the details of which can be consulted in the Supplementary Materials (Figures S1–S5). The normal probability plot indicated acceptable residual normality with minor deviations at the tails (Figure S1). The residuals vs. run plot confirmed the independence of the observations, as no systematic patterns were detected (Figure S2).

Slightly increased residual dispersion was observed at predicted response values with higher loops (Figure S3); however, no severe heteroscedastic behavior affecting model adequacy was identified, and the Box–Cox analysis confirmed that the selected log-transformation improves variance stability (Figure S4).

Four observations exhibited elevated Cook’s distance values (Figure S5). Again, these correspond to parameter combinations involving 15 loops. Combined with the slightly increased residual dispersion at these points, this highlights how heat-associated phenomena increase process variability and measurement uncertainty. Since these observations represent physically meaningful operating conditions and are structurally required by the factorial design, they were retained in the analysis. Additionally, these considerations strengthen the limitation on the number of loops to 15 and justify the statistical findings detailed in the subsequent section on multilayer substrate experiments.

### 3.2. Multilayer Substrate

The experimental conditions of the 2^3^ full factorial DoE are presented in Table 4.

**Figure 4:**
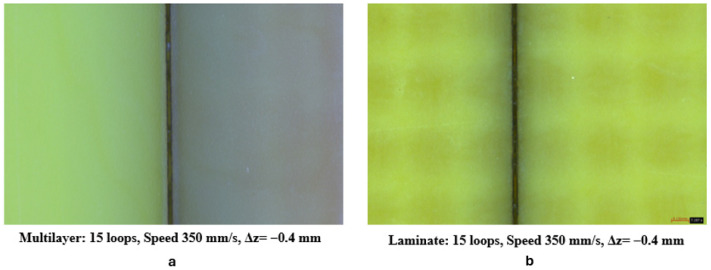
Heat-associated phenomena in multilayer (**a**) vs. laminate (**b**).

Additionally, preliminary tests showed that cutting a multilayer substrate—which contains not only double-sided laminates but also prepreg—results in reduced generation of heat-associated phenomena, such as charring and the diffusion of the heat-affected zone (Figure 4).

This is because prepreg typically exhibits a lower fiberglass reinforcement density than fully cured laminate (often around 40–50 wt% versus 60–70 wt% in laminates) which minimizes UV laser scattering, reduces thermal conduction pathways through the fibrous network, and limits resin decomposition hotspots, thereby suppressing heat-associated phenomena like charring and HAZ diffusion for cleaner, more precise cuts.

This physical explanation sets the maximum total number of loops to 16, exceeding the previously determined limit of 15. Additionally, the minimum number of loops was increased to 8 in accordance with the typical thicknesses of protective plugs.

By examining both simple laminate substrates and complex multilayer substrates, this study rigorously assesses whether prepreg inclusion induces substantive differences in the predictive models for UV laser penetration depth.

Again, for each DoE condition, five cuts were measured to ensure statistically significant results, and their means were used as the DoE response outputs, as shown in the following Table 5.

**Figure 5:**
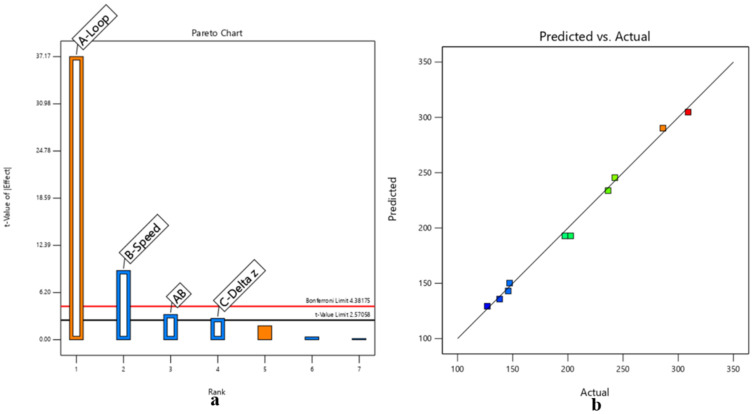

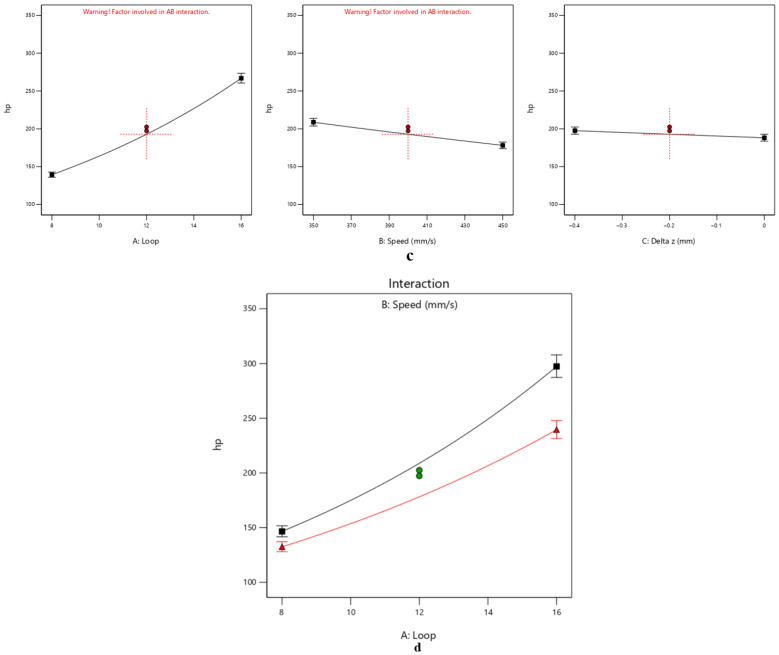
DoE-based analysis of UV laser plug removal in rigid–flex PCBs simulated with an FR-4-based multilayer, highlighting (**a**) the dominant parameters affecting removal selectivity, (**b**–**d**) process trends defining the stable micromachining window.

ANOVA was performed to assess the significance of the model terms, yielding the following results, detailed in Table 6 and Figure 5.

Analogously to the laminate substrate, the obtained model for hp in multilayer application can be expressed as a base-10 logarithmic law of the form ${log}_{10}(h_{p})$, where the statistically significant factors are:

- Loops, with a positive effect on the response.
- Speed, with a negative effect on the response.
- Loops–speed interaction, with a negative effect on the response.
- Focal position offset, with a negative effect on the response.

Based on the ANOVA results, speed–focal position interactions, loops–focal position interactions and third-order interactions were found to be statistically insignificant for predicting penetration depth (*p* > 0.05) and thus were excluded from the final predictive model. In this model as well, the most influential factor is the number of loops, contributing even more to the total variance in penetration depth (92.73%) than in the laminate predictive model. Consequently, the other parameters and interactions have a lesser relative impact compared to the laminate model but still significantly influence the ablation process. The considerations regarding the goodness of the model are the same as those presented in the section on the laminated substrate.

Additionally, the justification for the base-10 logarithmic form of the model was the same as explained in the previous section.

The generalized form of the model equation is:

(2) $${log}_{10}\left(h_{p}\right)=\alpha_{0}+\alpha_{1}*A-\alpha_{2}*B-\alpha_{3}*AB-\alpha_{4}*C$$

The underlying physical principles and key process considerations governing the multilayer model align with those established for the laminate substrate in the preceding analysis. Consequently, the process considerations for plug removal remain the same. This consistency suggests that the fundamental ablation mechanisms tend to remain stable despite the increased material complexity introduced by the prepreg layers.

The primary difference between the models lies in the significance and effects of their interactions: the loops–focal position interaction is insignificant in the multilayer case, while the loops–speed interaction exhibits a negative effect on penetration depth. This arises from the reduced heat-associated phenomena enabled by prepreg’s lower fiberglass reinforcement density, which minimizes UV laser scattering, curtails thermal conduction through the fibrous network, and suppresses resin decomposition hotspots. Consequently, ablation efficiency increases, yielding a more pronounced penetration depth reduction as scanning speed rises at constant loops, unlike the laminate substrate where residual heat sustains deeper cuts.

For this model as well, residual analysis was conducted to verify the underlying statistical assumptions, thereby confirming the adequacy of the predictive model and the validity of the ANOVA results. Details are available in the Supplementary Materials (Figures S6–S10). The diagnostics confirmed the adequacy of the model: the normal probability plot showed the linear alignment of residuals (Figure S6), residuals vs. predicted values indicated constant variance (Figure S7), the Box–Cox plot confirmed the variance stability (Figure S8), and no systematic patterns were observed in the residuals vs. run plot (Figure S9). No influential observations were detected based on Cook’s distance analysis (Figure S10).

It is interesting to evaluate the differences between the models obtained for the laminate and multilayer substrates. This was achieved by simulating both models at their boundary conditions to assess any significant differences between them. The analysis revealed that the predicted penetration depths differ by ∆= 6−17 μm, with the multilayer model consistently predicting deeper penetration (compared to the laminate model) under the same conditions.

The difference becomes more appreciable at higher numbers of loops, since the prepreg contributes less to heat-associated phenomena, thereby reducing interference and improving performance. Conversely, at lower numbers of loops, the difference is less pronounced, as thermal effects are minimal.

These considerations, combined with the residual diagnostics from both the laminate and multilayer models, highlight that the laminate model becomes less reliable at higher numbers of loops, whereas the multilayer model maintains its reliability. This stems from the stronger heat-associated phenomena in laminate applications, which lead to greater process variability and measurement uncertainty, as discussed in the previous section. In contrast, the presence of prepreg in the multilayer substrate results in less pronounced thermal effects and a more stable ablation process, yielding more predictable and controllable laser-cutting outcomes, along with more accurate measurements during testing.

Nevertheless, the laminate model provides a good approximation for complex stack-ups, since prepreg presence does not substantially alter UV laser–material interactions. In conclusion, using a laminate substrate to simulate multilayer rigid–flex PCB plug removal operations enables the rapid obtainment of preliminary process parameters with reduced experimental complexity and cost.

### 3.3. Model Validation

**Figure 6:**
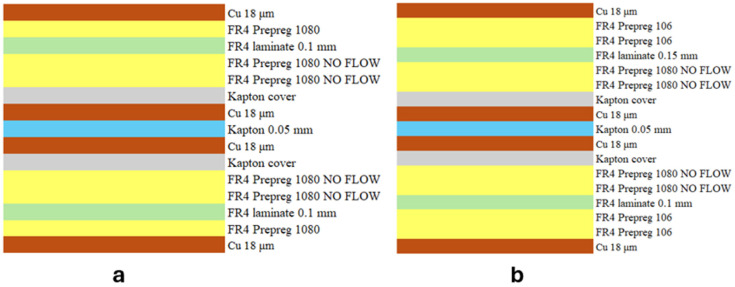
First (**a**) and second (**b**) stack-up used for model validation.

To validate the model’s accuracy and predictive capability, additional confirmation experiments were conducted to achieve compliant protective plug removal from PCBs’ flex sections. First, two stack-ups were selected (Figure 6).

Then, the parameters were determined using the multilayer model to calculate the optimal settings for loops, speed, and focal position offset required to achieve a compliant cut for the specific protective plug thicknesses defined in the selected stack-ups. These incorporate a 30 μm safety margin to prevent damage to the underlying Kapton cover while enabling safe plug removal without delamination risk. For these stack-ups, the protective plugs to be removed from stack-up 1 and stack-up 2 had thicknesses of 260 μm and 295 μm, respectively. The parameters obtained from the model were:

- Protective plugs stack-up n. 1:
  - Loops: 13.
  - Speed: 350 mm/s.
  - Δz = −0.4 mm.
- Protective plugs stack-up n. 2:
  - Loops: 15.
  - Speed: 350 mm/s.
  - Δz = −0.1 mm.

Subsequently, electrical integrity tests were performed on each PCB from the selected stack-ups to assess the adequacy of the optimized parameters and confirm no damage to underlying traces or the induction of short circuits. For this purpose, a flying probe system with a voltage of 5 V, a current of 25 mA, and a maximum acceptable resistance of 5 Ω was employed.

Electrical integrity tests were conducted on 48 PCBs from the first stack-up and 36 from the second, confirming no continuity issues or damage and achieving a 100% success rate, thereby demonstrating the model’s robustness and absence of overfitting.

## 4. Conclusions

This study successfully applied a comprehensive Design of Experiments framework, specifically two-level full factorial designs, to develop predictive mathematical models of the UV laser penetration depth in FR-4-based rigid–flex PCBs. This research systematically investigated the impact of key processing parameters—number of loops, scanning speed, and focal position offset—on material removal in both laminate and multilayer substrates, with the aim of enhancing process control and protective plug removal precision.

For the laminate substrate, the developed model, expressed as a base-10 logarithmic law, identified loops, speed, focal position offset, and their interactions as significant factors influencing penetration depth, with physically justified effects. Similarly, for multilayer substrates, a logarithmic model was established, where loops, speed, focal position offset, and the loop–speed interaction were found to be significant. A comparative analysis revealed that the multilayer model consistently predicted deeper penetration by 6–17 µm compared to the laminate model under identical conditions, particularly at higher numbers of loops, due to reduced heat-associated phenomena with prepreg, making the multilayer model more reliable. Therefore, performing experiments with a laminate substrate is suitable for reducing test complexity and costs. Despite this difference, the laminate model was found to offer a reasonable approximation for complex stack-ups.

Crucially, the models underwent rigorous validation through confirmation experiments focused on protective plug removal from PCB flex sections. These tests successfully determined optimal laser parameters for achieving compliant cuts with a 30 µm safety margin. The robustness and reliability of these models were unequivocally demonstrated by a 100% success rate in electrical integrity tests conducted on 84 PCBs (48 from the first stack-up and 36 from the second), confirming no damage to underlying traces or induction of short circuits. Although the exact coefficients of the predictive models are withheld for industrial confidentiality, their scientific validity is upheld via ANOVA verification and identification of key interaction mechanisms. This work provided a general understanding of how scanning speed, focal offset, and number of loops interact across FR-4 substrates. By reporting significance levels and interaction directions, it offers a reproducible framework for optimizing similar UV laser processes without disclosing specific constants. The main contributions—reported trends and laminate–multilayer comparisons—enable benchmarking for future rigid–flex PCB research.

In summary, this research provided a crucial tool for optimizing UV laser micromachining processes in rigid–flex PCB manufacturing. The proposed approach enables the definition of a robust UV laser micromachining window (which was defined by the parameter ranges presented in the previous DoEs) for protective plug removal in rigid–flex PCBs, supporting industrial implementation through quantitative process control rather than empirical tuning. It is important to emphasize that extrapolating beyond the previously cited parameter ranges may yield unreliable results. In conclusion, the derived predictive models significantly reduce the time required for parameter identification and minimize scrap generation, thereby contributing to enhanced efficiency, precision, and reliability in the production of advanced electronic components.

## Figures and Tables

**Table 1:** DoE matrix for laminate experimentation.

ID	A Loop	B Speed, mm/s	C Δz, mm
−1	5	350	−0.4
a	15	350	−0.4
b	5	450	−0.4
ab	15	450	−0.4
c	5	350	0
ac	15	350	0
bc	5	450	0
abc	15	450	0
CP	10	400	−0.2

**Table 2:** Measurement results for laminate experimentation.

ID	$\mathrm{Mean}\;\mathrm{of}\;h_{p}$, μm
−1	111.4
a	258.8
b	80.4
ab	247.6
c	111.6
ac	216.9
bc	78.2
abcd	182.8
CP	146.1

**Table 3:** ANOVA results for laminate experimentation.

Source	Sum of Squares	DF	Mean Square	F-Value	*p*-Value	% of Contribution
Model	0.3208	5	0.0642	320.72	<0.0001	/
A, Loops	0.2858	1	0.2858	1428.67	<0.0001	88.81
B, Speed	0.0190	1	0.0190	94.76	0.0002	5.89
C, Δz	0.0061	1	0.0061	30.26	0.0027	1.88
AB	0.0051	1	0.0051	25.64	0.0039	1.59
AC	0.0049	1	0.0049	24.28	0.0044	1.51
Lack of fit	0.0008	3	0.0003	2.89	0.2679	0.00
Pure Error	0.0002	2	0.0001	/	/	0.32
Total	0.3218	10	0.3855	/	/	100.00
$R^{2}=0.9969{\;\;\;\;\;R}_{adj}^{2}=0.9938\;\;\;\;\;R_{pred}^{2}=0.9683\;\;\;\;\;RMSE=4.60\;\mu m$						

**Table 4:** Measurement results for multilayer experimentation.

ID	A Loop	B Speed, mm/s	C Δz, mm
−1	8	350	−0.4
a	16	350	−0.4
b	8	450	−0.4
ab	16	450	−0.4
c	8	350	0
ac	16	350	0
bc	8	450	0
abc	16	450	0
CP	8	400	−0.2

**Table 5:** Measurement results for multilayer experimentation.

ID	$\mathrm{Mean}\;\mathrm{of}\;h_{p}$, μm
−1	147.2
a	309.0
b	138.2
ab	242.6
c	145.9
ac	286.1
bc	127.0
abcd	236.4
CP	197.3

**Table 6:** ANOVA results for multilayer experimentation.

Source	Sum of Squares	DF	Mean Square	F-Value	*p*-Value	% of Contribution
Model	0.1710	4	0.0427	370.69	<0.0001	/
A, Loops	0.1593	1	0.1593	1381.75	<0.0001	92.73
B, Speed	0.0095	1	0.0095	82.32	0.0008	5.52
C, Δz	0.0009	1	0.0009	7.80	0.0492	0.52
AB	0.0013	1	0.0013	10.89	0.0299	0.73
Lack of fit	0.0004	3	0.0001	2.08	0.4625	0.00
Pure Error	0.0001	1	0.0001	/	/	0.50
Total	0.1715	9	0.1712	/	/	100.00
$R^{2}=0.9973{\;\;\;\;\;R}_{adj}^{2}=0.9946\;\;\;\;\;R_{pred}^{2}=0.9820\;\;\;\;\;RMSE=4.06\;\mu m$						

## Data Availability

The original contributions presented in this study are included in the article/Supplementary Material. Further inquiries can be directed to the corresponding authors.

## Supplementary Materials

The following supporting information can be downloaded at: https://www.mdpi.com/article/10.3390/mi17030351/s1, Figure S1: Normal probability plot for laminate model. Figure S2: Residual vs run plot for laminate model. Figure S3: Residual vs. predicted plot for laminate model. Figure S4: Box-Cox plot for laminate model. Figure S5: Cook’s distance plot for laminate model. Figure S6: Normal probability plot for multilayer model. Figure S7: Residual vs. predicted plot for multilayer model. Figure S8: Box-Cox plot for multilayer model. Figure S9: Residual vs. predicted plot for multilayer model. Figure S10: Cook’s distance plot for multilayer model.
