# Supplementary material for: UV Laser Micromachining of FR-4-Based Rigid–Flex PCBs: Predictive Modeling of Penetration Depth Through Design of Experiments
Source: Micromachines (Basel). 2026 Mar 13;17(3):351. doi: 10.3390/mi17030351 (PMC13028538; doi:10.3390/mi17030351)
Supplement: Supplementary file 1 [file micromachines-17-00351-s001.zip › micromachines-4185241-supplementary.pdf]

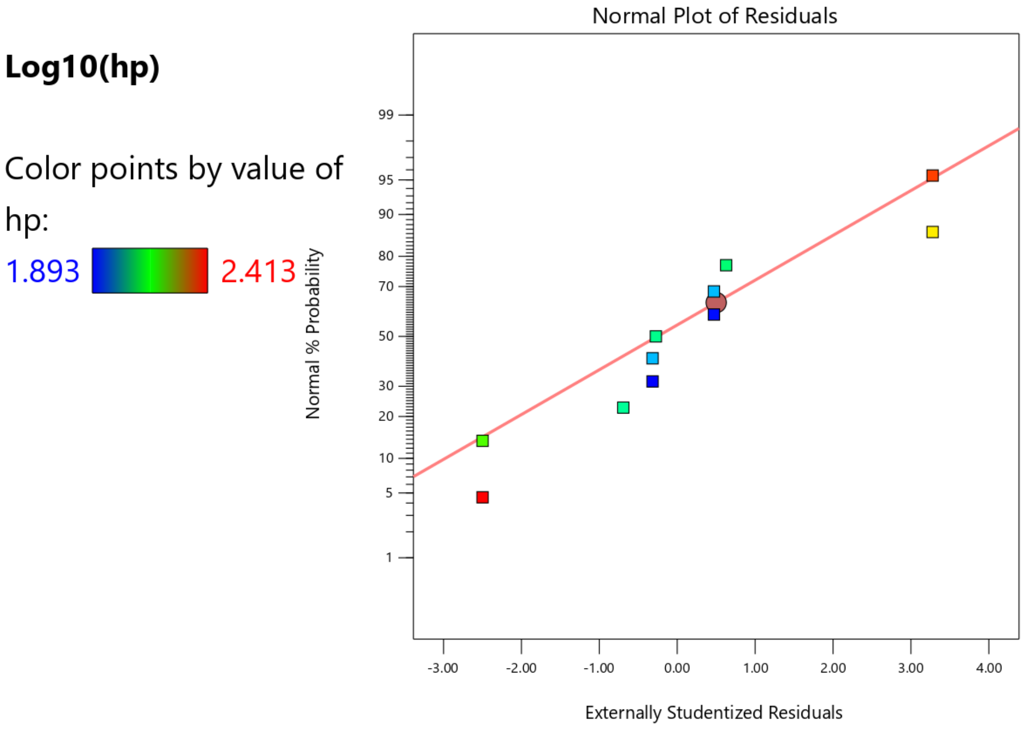

Figure S1. Normal probability plot for laminate model.

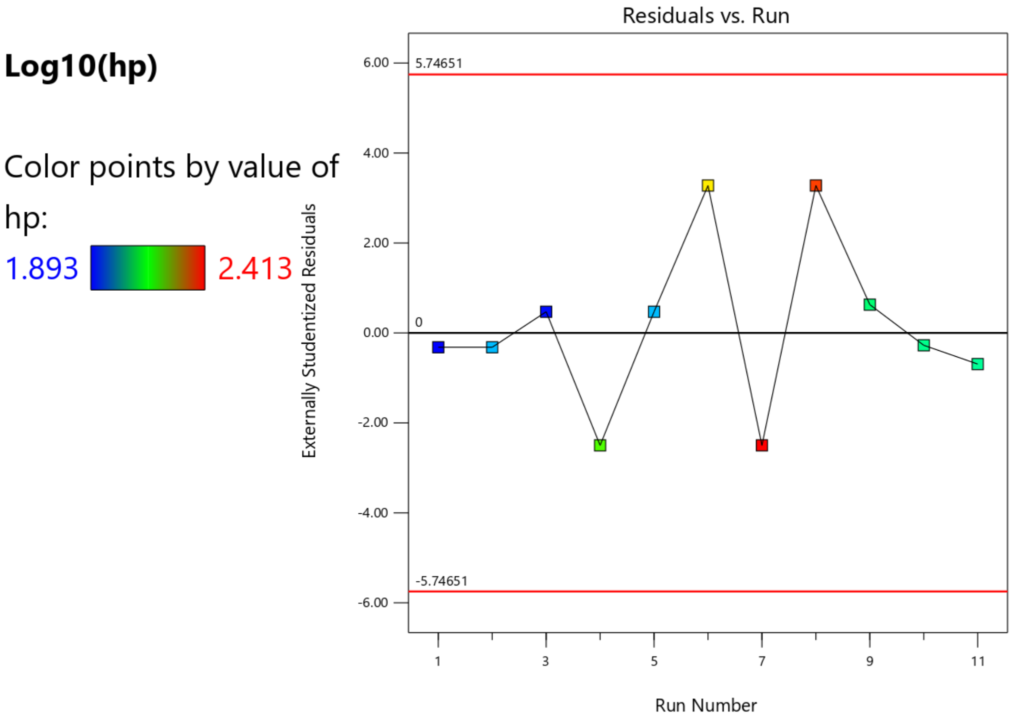

Figure S2. Residual vs run plot for laminate model.

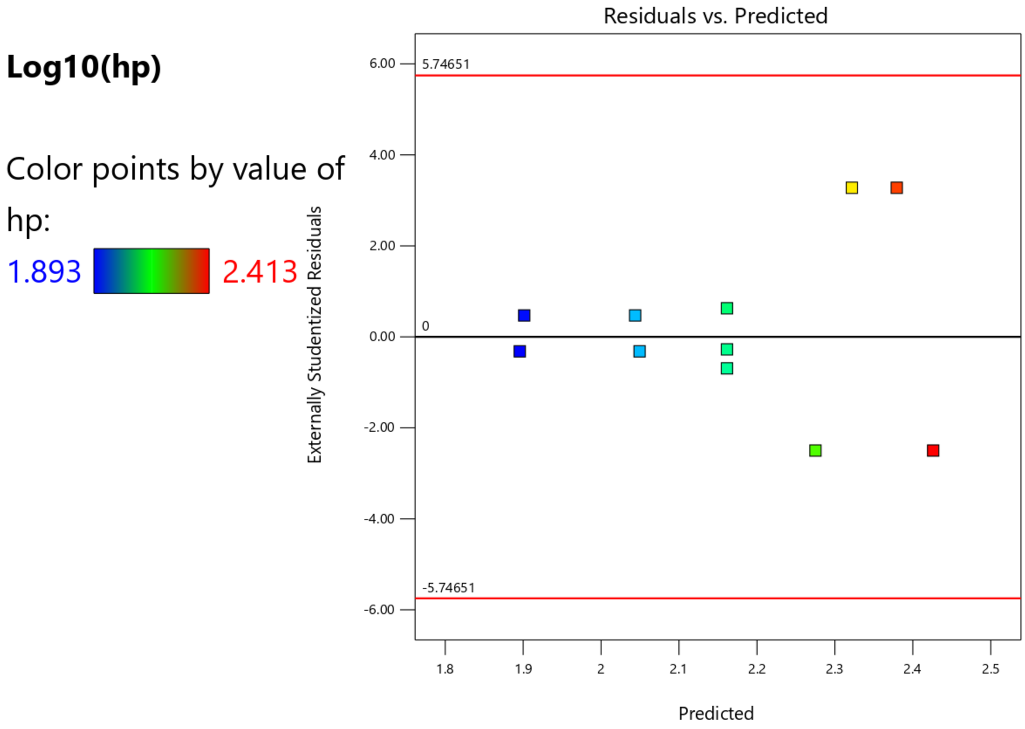

Figure S3. Residual vs predicted plot for laminate model.

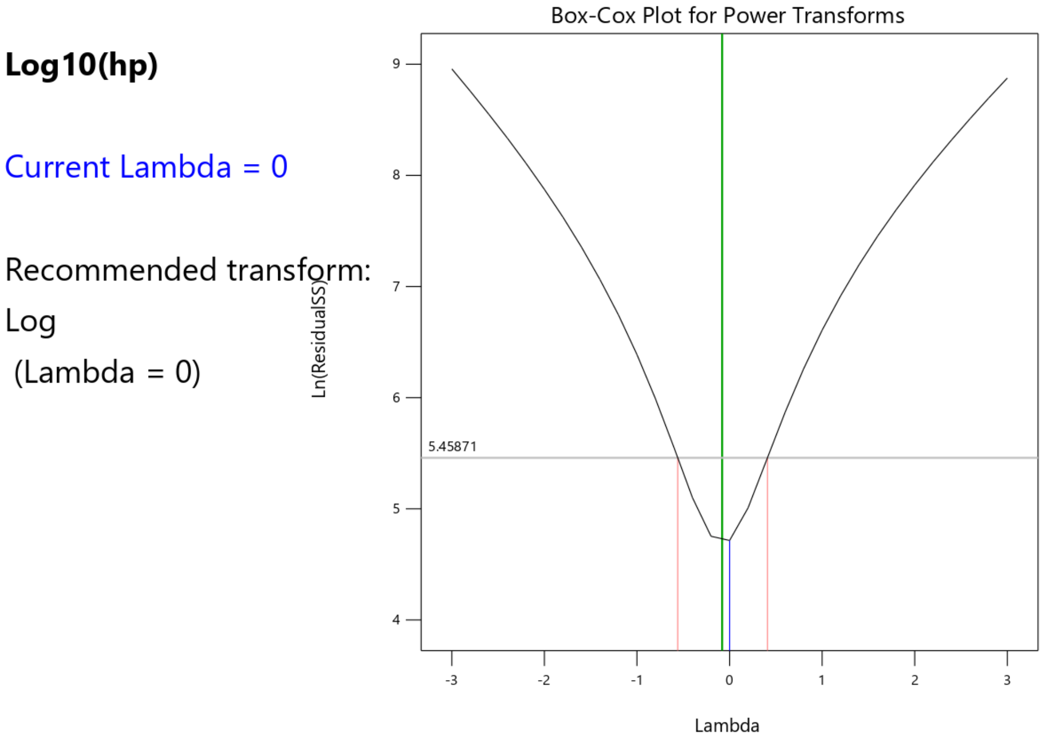

Figure S4. Box-Cox plot for laminate model.

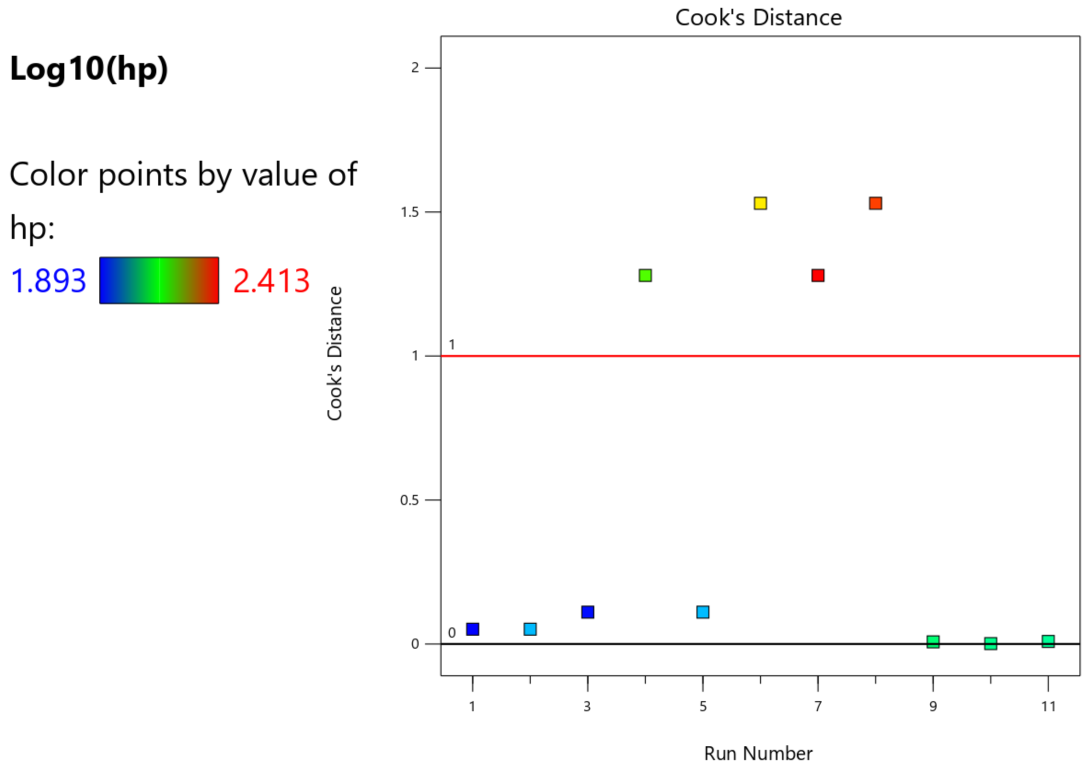

Figure S5. Cook's distance plot for laminate model.

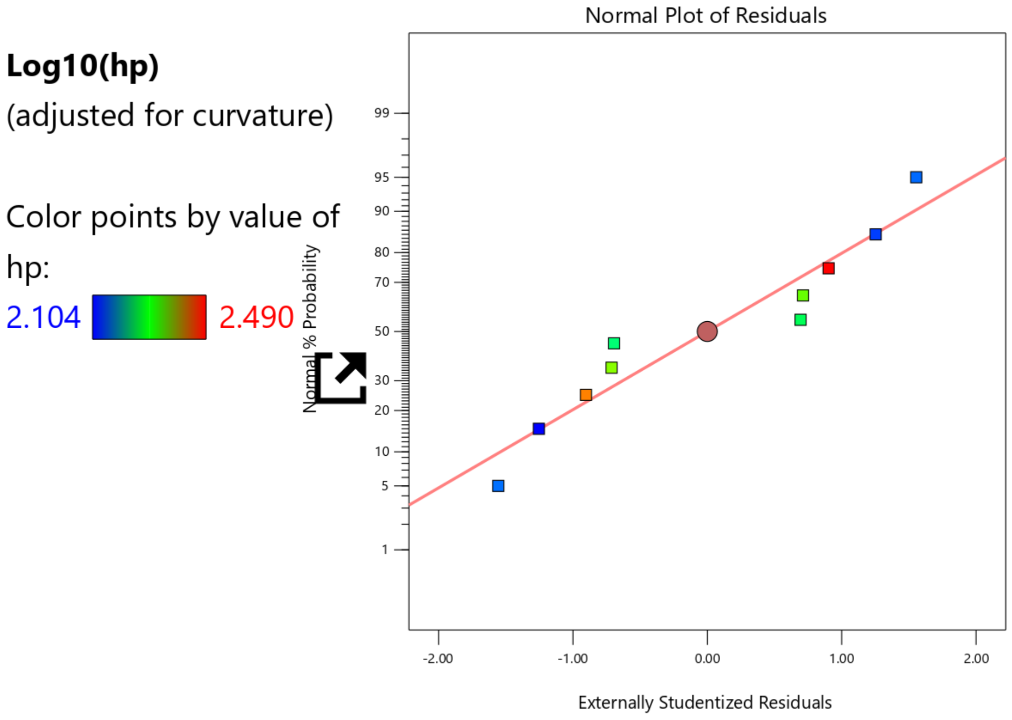

Figure S6. Normal probability plot for multilayer model.

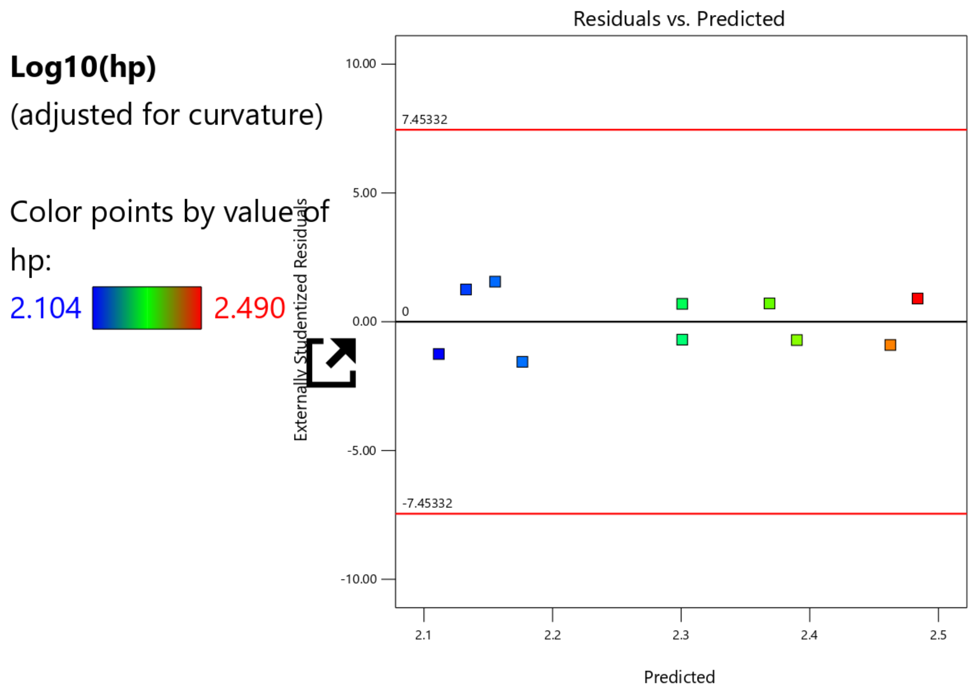

Figure S7. Residual vs predicted plot for multilayer model.

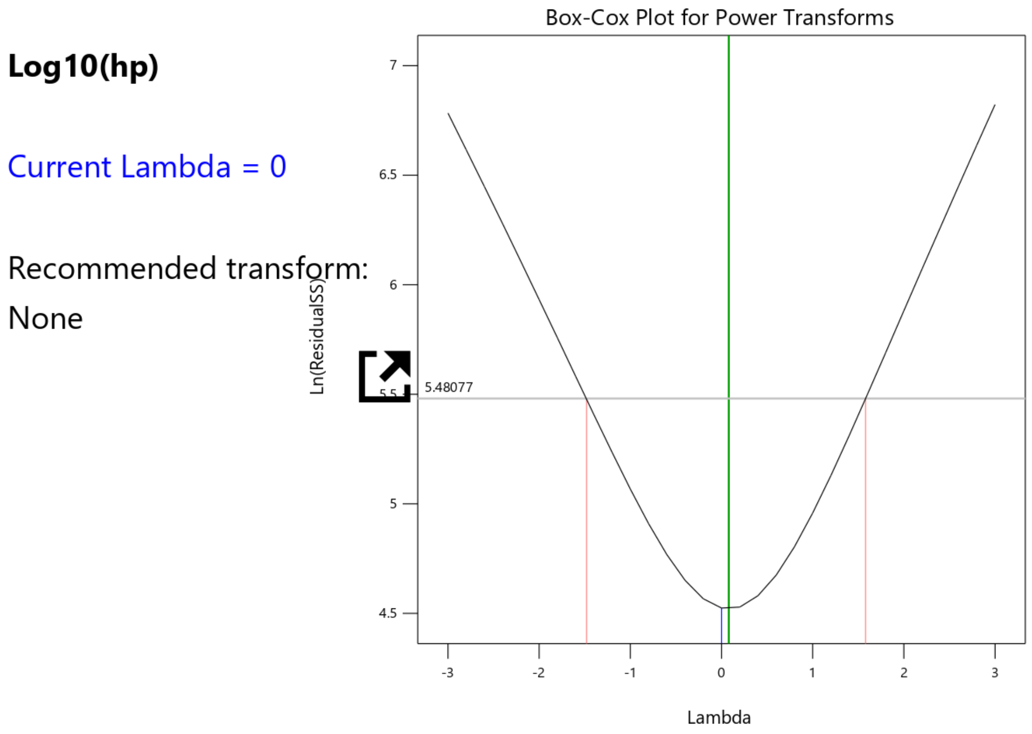

Figure S8. Box-Cox plot for multilayer model.

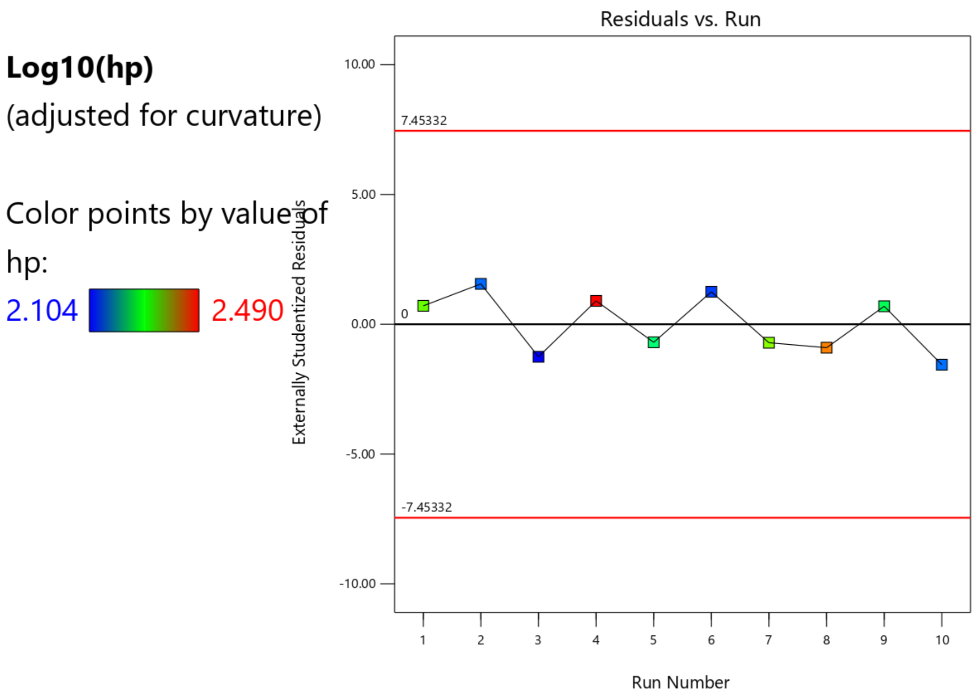

Figure S9. Residual vs predicted plot for multilayer model.

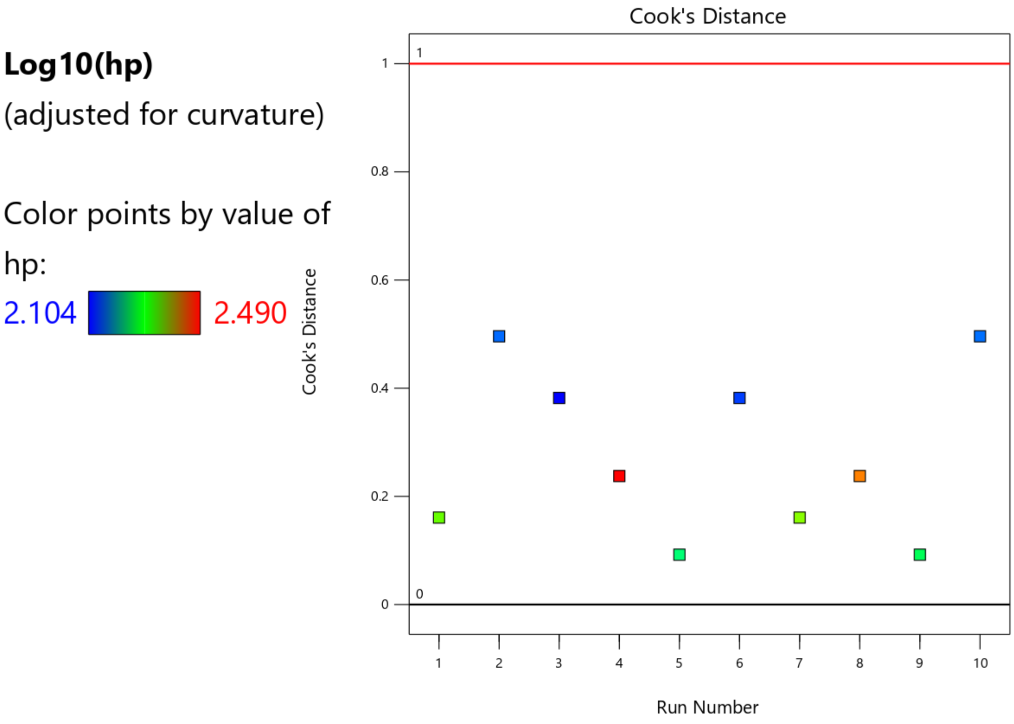

Figure S10. Cook's distance plot for multilayer model.
